# The cytokine interleukin-26 as a biomarker in pediatric asthma

**DOI:** 10.1186/s12931-016-0351-6

**Published:** 2016-03-31

**Authors:** Jon R. Konradsen, Björn Nordlund, Bettina Levänen, Gunilla Hedlin, Anders Linden

**Affiliations:** Clinical Immunology and Allergy Unit, Department of Medicine Solna, Karolinska Institutet and Karolinska University Hospital, SE-171 76 Stockholm, Sweden; Centre for Allergy Research, Karolinska Institutet, SE-171 77 Stockholm, Sweden; Astrid Lindgren Children’s Hospital, Karolinska University Hospital Solna, SE-171 76 Stockholm, Sweden; Department of Women’s and Children’s Health, Karolinska Institutet, SE-171 76 Stockholm, Sweden; Unit for Lung and Airway Research, Institute of Environmental Medicine, Karolinska Institutet, PO Box 210, SE-17177 Stockholm, Sweden; Lung Allergy Clinic, Karolinska University Hospital Solna, SE-171 76 Stockholm, Sweden

**Keywords:** Asthma, Children, Eosinophil, FENO, IL-26, Neutrophil, Sputum

## Abstract

In this pilot study, we examined associations between local interleukin (IL)-26, disease severity and biomarkers of Th2-mediated inflammation in a well-defined cohort of pediatric patients (14 years median age, 41 % females) with controlled (*n* = 28) or uncontrolled (*n* = 48) asthma. Sputum IL-26 protein concentrations (ELISA) reflected disease control in patients without local (low exhaled nitric oxide) or systemic (low blood eosinophils) signs of eosinophilic inflammation. Moreover, sputum-IL-26 concentrations correlated with those of blood neutrophils. Our study indicates that IL-26 is a potential biomarker of disease severity in pediatric asthma without signs of Th2-mediated inflammation.

## Introduction

Dear Editor,

Up to 20 % of children with severe asthma have no signs of eosinophilic inflammation [1] and these pediatric patients respond poorly to inhaled corticosteroids and anti-IgE, pharmacotherapy that was originally designed to target severe Th2-mediated inflammation [2]. For these patients, there is a need to increase the understanding of the immunological events underlying the disease, to establish biomarkers for improved diagnosis and monitoring, as well as to identify potential targets for therapy [2].

The presumed Th17 cytokine interleukin (IL)-26 is an intriguing member of the IL-10 family; one that is involved in several chronic inflammatory disorders and can exert both pro- and anti-inflammatory actions, depending upon the setting [3]. However, there is currently no conclusive information on the involvement of IL-26 in the pathogenesis of asthma or any other chronic inflammatory airway disorder [4]. A recent study on healthy human subjects indicates that this cytokine is produced by Th17 cells, as well as by other leukocytes, and that IL-26 contributes to the mobilization of neutrophils in the airways during activation of pulmonary host defense by endotoxin [5]. Given these facts, we hypothesized that local IL-26 is associated with severe disease in pediatric asthma lacking signs of eosinophilic inflammation.

## Methods

To address our hypothesis, we utilized data from a previously well-characterized cohort of school-age children (*n* = 76) with severe uncontrolled (uncontrolled) or persistent controlled (controlled) asthma [6]. Briefly, uncontrolled asthma was diagnosed in a child with reduced asthma control despite treatment with high doses of inhaled corticosteroids (≥800 μg budesonide equivalent *per* 24 h), whereas controlled asthma was defined as children having an acceptable asthma control with a low to moderate daily dose of inhaled corticosteroids (100–400 μg budesonide equivalent *per* 24 h). The patients were recruited as previously described [6], after approval by the Regional Ethics Review Committee in Stockholm (Stockholm) and after informed consent from each patient or parent, in full accordance with the Helsinki declaration.

We quantified IL-26 protein concentrations in the airways in relation to asthma severity, blood eosinophils (B-EOS), exhaled nitric oxide (FENO) and blood neutrophils (B-NEUTRO). Specifically, we utilized cell-free samples of induced sputum [7] for the measurement of IL-26 protein concentrations (ng/mL) with ELISA (Cusabio Biotech®) [5].

## Results

Among our included pediatric patients, the median age (range) was 13.7 years (7–19), with 41 % being females. The 48 patients with uncontrolled asthma had a reduced score on the asthma control test (17 versus 23, *p* < 0.001), and a lower FEV_1_ (82 % versus 90 % predicted, *p* = 0.04) despite receiving a higher dose of inhaled corticosteroids (800 μg versus 320 μg of budesonide, *p* < 0.001) compared to the 28 children with controlled asthma. In addition, the children with uncontrolled asthma had higher concentrations of B-EOS (0.4 versus 0.2, *p* = 0.02) and B-NEUTRO (3.2 versus. 2.6, *p* = 0.03) than those with controlled asthma.

In the entire cohort, the median FENO levels were 18.3 p.p.b and the median B-EOS concentration was 0.3×10^9^/L. These median values were used to categorize children into groups with high and low levels of these biomarkers of Th2-mediated inflammation, independently of the predefined severity classification.

**Fig. 1:**
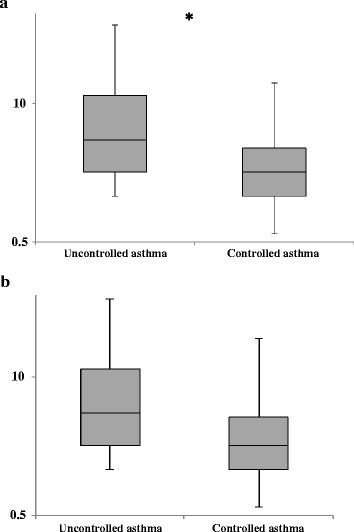
Median concentrations (logarithmically transformed) of IL-26 (ng/mL) in induced sputum from children with uncontrolled versus controlled asthma and **a** low concentrations of eosinophils in blood (≤0.3 × 10^9^/L) or **b** low levels of exhaled nitric oxide (≤ 18.3 p.p.b)

Among children with low concentrations B-EOS (≤0.3*10^9^/L), we observed higher concentrations of IL-26 in uncontrolled compared to controlled asthma (Fig. 1a & Table 1). Furthermore, we found a corresponding association between asthma control and IL-26 concentrations among children with low levels of FENO (Fig. 1b & Table 1). A trend towards an association between asthma control and IL-26 concentrations in the entire cohort was observed, but it was not statistically significant (Table 1). Furthermore, when investigating children with high concentrations of B-EOS and high levels of FENO, the difference in distribution of IL-26 between children with uncontrolled and controlled asthma was less pronounced (Table 1).

**Table 1:** Interleukin-26 in the airways of children with uncontrolled versus controlled asthma

	IL-26 (ng/ml) Uncontrolled asthma	n	IL-26 (ng/ml) Controlled asthma	n	*p*-value*
All patients	4.4 (1.9–14)	48	2.4 (1.3–4.5)	28	0.077
Low B-EOS (≤ 0.3×10^9^/L)	4.6 (2.3–13)	27	2.3 (1.3–4.5)	22	0.038*
High B-EOS (>0.3×10^9^/L)	4.1 (1.7–16)	21	2.9 (1–31)	5	0.61
Low FENO (≤18.3 p.p.b.)	4.1 (2.2–16)	23	2.0 (1.2–3.6)	13	0.055
High FENO (>18.3 p.p.b)	5.0 (1.8–13)	25	2.7 (1.4–24)	15	0.54

*Footnotes*. The IL-26 concentrations are presented as medians with inter-quartile ranges

The median values in the entire cohort of the respective biomarker were used to define the cut-off between high and low levels of the various biomarkers. *B-EOS* blood eosinophils, *FENO* the fraction of nitric oxide in exhaled air, in parts per billion. *Independent samples, analyzed by Mann–Whitney U-test (SPSS® version 20)

Finally, we found a weak but statistically significant correlation (the Pearson test: *r* = 0.27, *p* = 0.018, *n* = 76) between the concentrations of B-NEUTRO and IL-26 in the entire cohort of pediatric patients with asthma and this correlation tended to be stronger in the patients with low levels of FENO (*r* = 0.35, *p* = 0.036, *n* = 36). There was no evident correlation between IL-26 concentrations and age, gender, height or dose of inhaled corticosteroids.

## Discussion

Our study is the first to associate local IL-26 protein concentrations in the airways with a certain clinical phenotype of pediatric asthma. The fact that IL-26 protein concentrations are increased in sputum from pediatric patients with uncontrolled asthma but without signs of Th2-mediated inflammation forwards IL-26 as a potential, novel biomarker of disease severity in this phenotype of asthma. These findings are also supported by a positive correlation between local IL-26 and systemic neutrophils (ie. B-NEUTRO); a correlation in line with a mechanistic link between IL-26 and the mobilization of neutrophils as recently indicated in human airways [5, 8].

One of the effects of treatment with inhaled corticosteroid is an inhibition of the production of Th2 cytokines such as IL-4 and IL-5, which subsequently attenuate eosinophilic recruitment and the release of toxic granulae proteins [9]. The observed clinical effects are an improvement of symptoms and pulmonary function and a reduced rate of asthma exacerbations [10]. It is widely accepted that increased concentrations of blood eosinophils and high levels of FENO indicate systemic and local eosinophilic inflammation, respectively [11, 12]. In addition, it is known that low levels of these established biomarkers are associated with a reduced sensitivity to inhaled steroids [13, 14]. Given that our findings are compatible with an increase in local IL-26 being associated with inflammation not mediated by Th2-related mechanisms; this observation forwards the clinically relevant question whether IL-26 is also linked to low sensitivity to inhaled steroids in pediatric patients with non-allergic asthma? New studies are required to address this possibility; a possibility that has implications for the estimated 13 % of children with severe asthma displaying excess neutrophil mobilization [1].

Even though the size of our study material was modest, a particular strength of our current analysis is the standardized and detailed characterization of each individual patient; making it possible to identify various subgroups within this cohort. We think that this facilitated the discovery that IL-26 provides a potentially clinically relevant signal in patients without signs of local or systemic eosinophilic inflammation.

## Conclusion

Our pilot study demonstrates that local IL-26 bears potential as a biomarker of disease severity in a clinical phenotype of pediatric asthma that is perpetuated by other than Th2-related mechanisms.

## Abbreviations

B-EOS: blood eosinophils
B-NEUTRO: blood neutrophils
FENO: the fraction of nitric oxide in exhaled air
FEV_1_: forced expiratory volume during 1 s
IL: interleukin
Th: T helper
